# Nutrition at the Intersection between Gut Microbiota Eubiosis and Effective Management of Type 2 Diabetes

**DOI:** 10.3390/nu16020269

**Published:** 2024-01-16

**Authors:** Sevag Hamamah, Oana C. Iatcu, Mihai Covasa

**Affiliations:** 1 Department of Basic Medical Sciences, College of Osteopathic Medicine, Western University of Health Sciences, Pomona, CA 91766, USA; sevag.hamamah@westernu.edu; 2 Department of Biomedical Sciences, College of Medicine and Biological Science, University of Suceava, 720229 Suceava, Romania

**Keywords:** gut bacteria, macronutrients, micronutrients, food groups, insulin resistance

## Abstract

Nutrition is one of the most influential environmental factors in both taxonomical shifts in gut microbiota as well as in the development of type 2 diabetes mellitus (T2DM). Emerging evidence has shown that the effects of nutrition on both these parameters is not mutually exclusive and that changes in gut microbiota and related metabolites such as short-chain fatty acids (SCFAs) and branched-chain amino acids (BCAAs) may influence systemic inflammation and signaling pathways that contribute to pathophysiological processes associated with T2DM. With this background, our review highlights the effects of macronutrients, carbohydrates, proteins, and lipids, as well as micronutrients, vitamins, and minerals, on T2DM, specifically through their alterations in gut microbiota and the metabolites they produce. Additionally, we describe the influences of common food groups, which incorporate varying combinations of these macronutrients and micronutrients, on both microbiota and metabolic parameters in the context of diabetes mellitus. Overall, nutrition is one of the first line modifiable therapies in the management of T2DM and a better understanding of the mechanisms by which gut microbiota influence its pathophysiology provides opportunities for optimizing dietary interventions.

## 1. Introduction

The human gastrointestinal (GI) tract harbors trillions of gut microbiota, comprising about 500–1000 different bacterial species, which collectively weigh approximately 1–2 kg [1,2]. Analysis of the human microbial composition has shown that the gut microbiota of adults contains six phyla, with 90% of these bacterial species belonging to the phyla Bacteroidetes and Firmicutes, while the phyla Actinobacteria, Proteobacteria, Fusobacteria, and Verrucomicrobia make up the rest [3]. The balance of the gut microbiota, namely eubiosis, is important in maintaining health and preventing diseases [4]. It has been well documented that the profile of the gut microbiota is unique to each individual host, and its composition is influenced by a variety of factors, resulting in taxonomical shifts in microbial species throughout a person’s lifetime [5,6]. This includes both extrinsic factors such as lifestyle, stress, medication, diet, and disease status, as well as intrinsic factors, such as genetics, immune, or metabolic factors [5,7], with extrinsic factors having the greatest effect on gut microbiota [7]. The influence of gut microbiota on homeostatic processes in the human body is multifaceted, with important roles in modulating metabolic processes [8], regulating immune responses [9], and maintaining overall host health [5]. More specifically, the effects of gut microbiota have been linked to a myriad of non-communicable diseases including type 2 diabetes mellitus (T2DM), dyslipidemia, obesity, and Parkinson’s disease [10,11,12,13], to name a few. In particular, T2DM, a multifactorial chronic metabolic condition that is characterized by hyperglycemia, lipid imbalance, and insulin resistance [14], remains a major global health threat affecting approximately 6% of the world’s population [15] and contributing significantly to the worldwide socio-economic burden [10]. The prevalence of T2DM continues to increase, with an estimated 530 million individuals being affected by the disease, of which 22% are above the age of 70 [15]. Therefore, gaining a deeper understanding of the factors and mechanisms controlling hyperglycemia and insulin resistance is critical in the prevention, management, and effective therapeutic interventions of diabetes.

The gut microbiota has been long recognized as a key component in regulating host health and specific bacteria have been causally linked with the onset and progression of diseases, including diabetes. The extent of the effects of gut bacteria on T2DM has been attributed to both taxonomical shifts in gut microbiota as well as the differential production of important gut metabolites including short-chain fatty acids (SCFAs), bile acids (BAs), and amino acids (AAs), which are shown to contribute to or protect against hyperglycemia and insulin resistance [10]. For example, bacterial species belonging to the genera *Bifidobacterium* and *Lactobacillus*, which are significant producers of SCFAs, have been correlated with a reduction in HbA1c serum levels [16]. Similarly, the altered absorption of SCFAs and BAs have been observed in patients with T2DM as a result of increased gut barrier permeability induced by the dysbiosis of gut flora [17]. Importantly, nutrition has been shown to be the primary modifiable factor of gut microbiota remodeling and the development of T2DM, with various diets, food groups, macronutrients, and micronutrients exerting different effects on its composition [18,19]. For example, beneficial diets such as the Mediterranean diet, primarily composed of plant-based products, are inversely related with HgbA1c levels, waist circumference, and insulin resistance [20], while animal-based product diets promote opposite effects [21]. Further, the type of food as well as the macronutrient and micronutrient composition of the diet exert distinct effects on gut microbiota and related metabolites, with major consequences on mechanisms regulating hyperglycemia and insulin resistance [21,22,23]. This review describes the key role of nutrients at the intersection between gut microbial eubiosis and the development of T2DM. It presents changes in the gut microbiota composition profile of individuals with T2DM and how specific gut bacteria and related metabolites contribute to, or safeguard against, diabetes. Further, the effects of various macronutrients and micronutrients on the microbiota–T2DM relationship and the impact of the common food groups on the gut microbial composition and T2DM are discussed.

## 2. Influence of Gut Microbiota on Hyperglycemia, Insulin Resistance, and T2DM

Over the past several years, numerous studies have linked gut microbiota and T2DM, with factors such as systemic inflammation through the production of lipopolysaccharides (LPSs) [24], changes in gut membrane permeability, and bile acid metabolism [25] all playing significant roles in the degree of insulin resistance in the host [1,10]. Gut bacteria have been associated with glucose intolerance as germ-free mice show differential resistance against high fat diet-induced insulin resistance and adiposity [26,27,28]. More specifically, studies evaluating conventional gut microbial composition in T2DM showed important trends in taxonomical shifts in gut bacteria that may have strong associations with the pathogenesis of condition. For example, an increase in the Firmicutes-to-Bacteroidetes ratio has been linked to conditions associated with low-grade inflammation such as obesity and T2DM [29,30]. Further, the dysbiosis in T2DM has been characterized by a decrease in butyrate-producing bacterial species, mainly *Roseburia intestinalis*, *Bifidobacterium* spp., *Akkermansia* spp., and *Faecalibacterium prausnitzii*, and an increase in the abundance of unfavorable bacteria such as *Clostridium clostridioforme*, *Clostridium hathewayi*, *Clostridium ramosum*, *Clostridium symbiosum*, *Bacteroides caccae*, *Escherichia Coli*, *Eggerthella* spp., *Fusobacterium,* and mucin-degrading bacterial genera, *Ruminococcus* [31,32,33,34]. Studies linking *Lactobacillus* spp. with type 2 diabetes have been inconsistent [31].

**Figure 1:**
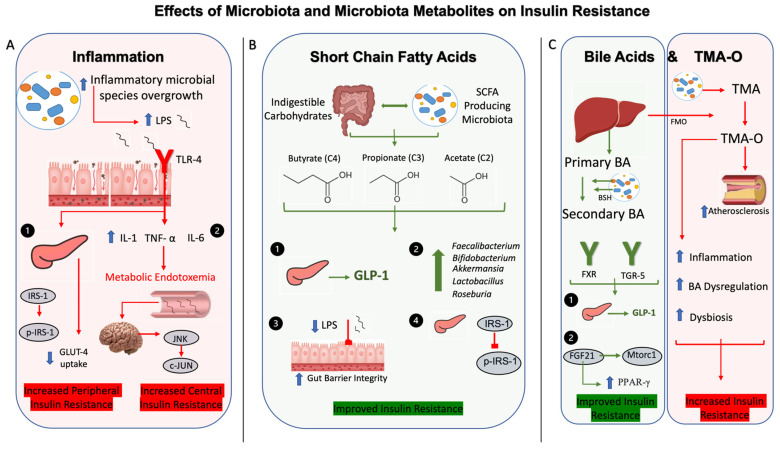
Effects of microbiota and metabolites on glucose homeostasis and insulin resistance. (**A**) The overgrowth of inflammatory microbial species, particularly Gram-negative bacteria, promotes increased LPS and gut permeability. LPS/TLR-4 binding leads to IRS-1 phosphorylation and decreased GLUT-4 uptake, thereby increasing serum blood glucose and worsening peripheral insulin resistance. LPS/TLR-4 binding contributes to pro-inflammatory cytokine release and metabolic endotoxemia. Metabolic endotoxemia contributes to the hypothalamic JNK cascade and c-JUN activity. c-JUN interacts with IRS-1 to promote central insulin resistance. (**B**) SCFA-producing microbiota have the capability to enzymatically catalyze fermentation reactions to produce SCFA; butyrate, propionate, and acetate. SCFAs enhance GLP-1 secretion, increase relative abundances of beneficial bacteria, improve gut barrier integrity, and reduce phosphorylation of IRS-1 to improve insulin resistance. (**C**) Primary bile acids are created in the liver, which are further converted by gut microbiota into secondary bile acids. Secondary bile acids bind to their receptors, FXR and TGR5, to enhance GLP-1 secretion and stimulate the FGF21 pathway to increase PPAR-γ, improving insulin resistance. At the same time, they promote the activation of the Mtorc1 pathway, which uncouples IRS-1 and promotes insulin resistance. Gut microbiota produce TMA, which are converted by FMO in the liver to TMA-O. TMA-O contributes to atherosclerosis, inflammatory processes, bile acid dysregulation, and dysbiosis leading to increased insulin resistance. Abbreviations: LPS, Lipopolysaccharides; TLR-4, Toll-like receptor 4; IL-1, interleukin-1; TNF-α, Tumor necrosis factor alpha; IL-6, interleukin-6; IRS-1, Insulin receptor substrate 1; p-IRS-1, phosphorylated insulin receptor substrate 1; GLUT-4, glucose transport 4; JNK, Jun amino terminal kinase; C4, 4 carbon; C3, 3 carbon; C2, 2 carbon; GLP-1, Glucagon-like peptide 1; BA, bile acid; FGF21, Fibroblast Growth Factor 21; Mtorc1, Mammalian target of rapamycin complex 1; PPAR-γ, Peroxisome proliferator-activated receptor gamma; FMO, Flavin-containing monooxygenase; TMA, Trimethylamine N; TMA-O, Trimethylamine N-oxide.

The collective taxonomical shifts in gut microbiota composition are associated with increased gut and systemic inflammation, further contributing to the pathogenesis of T2DM [35]. For example, an increase in the pathogenic Gram-negative bacteria leads to the release of lipopolysaccharides (LPSs), which is known to activate toll-like receptor 4 (TLR4) in adipocytes, promoting inflammatory signaling and cytokine expression [36]. Previous data have shown that LPS binding to TLR4 is associated with insulin resistance, because mice lacking TLR4 are protected from suppressed insulin signaling and insulin-mediated changes in glucose metabolism [37]. Further studies have shown that adipocytes expressing TLR4 promote the induction of pro-inflammatory cytokines, particularly IL-6 and IL-8, which decreased insulin-induced glucose uptake through downregulation of insulin-receptor substrate 1 (IRS-1) and glucose transporter 4 (GLUT4) [38]. Serine kinases, such as c-Jun-N-terminal Kinase (JNK) and inhibitor of nuclear factor kappa-B kinase subunit beta (IKKβ), contribute to insulin resistance through phosphorylation of IRS-1 [39,40]. Additionally, the mechanism behind increased insulin resistance, adiposity, and lipid abnormalities is also thought to be due to the LPS-induced increase in gut permeability by the reduced expression of tight junction proteins, primarily zonula occludens-1 and minimally to claudin and occluden [41]. The impaired gut barrier integrity leads to the translocation of LPS into the bloodstream and the development of metabolic endotoxemia [42]. This, in turn, contributes to a sustained low-grade inflammation, via central insulin resistance and activation of the hypothalamic c-Jun N-terminal Kinase (JNK) cascade [43]. Taken together, these findings provide strong evidence for the influence of bacteria and its byproducts in altered insulin sensitivity (Figure 1).

The gut microbiota byproducts have been shown to exert generally protective effects on hyperglycemia and insulin resistance. Among them, SCFAs, the enzymatically degraded end-product of the anaerobic fermentation reactions of indigestible complex carbohydrates [44] such as butyrate, propionate, and acetate, are the most metabolically important [45]. For example, the oral supplementation of butyrate in a diabetic rodent model significantly decreased serum hemoglobin A1c, LPS, and pro-inflammatory cytokine levels, while concomitantly improving gut integrity through the measurement of intracellular adhesion molecules [46]. Importantly, these findings were accompanied by an increased Firmicutes-to-Bacteroidetes ratio, which correlates with previous data showing the interplay between SCFA production and increases in gut microbial diversity [47]. Similar findings have been demonstrated with the administration of a butyric acid derivative attenuating LPS-induced inflammation and insulin resistance with decreased phosphorylated IRS-1 measured in mouse adipocytes [48]. This involves the activated protein kinase (AMPK)-dependent signaling, with beneficial effects including decreased inflammation, the survival of β cells, inhibition of insulin resistance, and promotion of glucose metabolism and uptake [49]. Of note, acetate is also shown to have similar benefits, with increased AMPK signaling activity in the liver leading to hypoglycemic effects [49]. Similarly, SCFAs have been shown to mitigate inflammatory processes, specifically through reprogramming the metabolic activity of T lymphocytes [50]. For example, propionate enhanced the expression of T regulatory cells, particularly Th17 helper cells and interleukin 10 (IL-10), ameliorating the negative effects of high-fat diet feeding [51]. T regulatory cells in adipocytes are shown to reduce adipose tissue inflammation and improve insulin resistance, further supporting the anti-inflammatory effects of SCFAs on metabolic disease [52]. In addition to their anti-inflammatory effects and improvement of hyperglycemia, SCFAs also exert antidiabetic effects through insulin secretion in a glucose-dependent manner by stimulating the secretion of glucagon-like peptide 1 (GLP-1) via the free fatty acid receptors, FFAR2 and FFAR3, located on enteroendocrine cells [53]. Probiotic administration increased the levels of both SCFAs and SCFA-producing bacterial species, while decreasing pathogenic Escherichia coli and LPS [54]. Interestingly, it has also been shown that metformin, one of the first-line treatments for T2DM, promotes the abundance of SCFA-producing gut microbiota, which is correlated to the secretion of GLP-1 [55]. Therefore, SCFAs play an integral role in ameliorating T2DM through improving inflammation, activating important signaling pathways and the modulation of gut peptides (Figure 1).

In addition, crosstalk between bile acids and gut microbiota play significant roles in the development or protection against hyperglycemia and insulin resistance [25]. Studies have shown that gut microbiota are involved in enzymatically converting primary bile acids into secondary bile acids [55], through the expression of bile salt hydrolase activity [25]. Secondary bile acid binding to the farsenoid X receptor (FXR) and Takeda G-protein coupled receptor 5 (TGR5) are shown to restructure gut microbiota and influence markers of T2DM [56]. For example, TGR5 activation can enhance pancreatic and liver function, leading to enteroendocrine L-cell-mediated GLP-1 release and improved insulin resistance [57]. FXR agonists improve insulin resistance in diabetic animal models [58]; however, other studies shown that FXR deficiency has a similar effect [59,60]. Further, bile acids have been shown to increase insulin sensitivity through the fibroblast growth factor (FGF) activity, with FGF21 signaling found to have beneficial effects both in the liver and in adipose tissue, through the decreased activation of the mammalian target of rapamycin complex 1 (Mtorc1) pathway [61] and increased activation of peroxisome proliferator-activated receptor γ (PPARγ) [62], respectively. Specifically, signaling via the Mtorc1 pathway promotes the serine phosphorylation of IRS-1 (p-IRS-1), a known marker of insulin resistance [61]. Conversely, the activation of PPARγ improves insulin-mediated skeletal muscle glucose uptake and hepatic glucose production to enhance insulin sensitivity [63]. Overall, it is evident that these receptors and pathways influenced by the enterohepatic circulation of bile acids and gut microbiota are heavily implicated in glucose homeostasis and insulin sensitivity (Figure 1).

## 3. Influence of Nutrition in Modulating Gut Microbiota and Markers of T2DM

Nutrition plays a critical role in the intricate relationships between gut microbiota and the pathophysiology of T2DM, and it is the key common factor when considering microbiota altering interventions to improve hyperglycemia and insulin resistance [64]. Nutrition shapes the gut microbiota, and it accounts for over 20% of the inter-individual microbiome variability in humans and 50% in mouse models [65,66]. Therefore, identification of different diets, macronutrients, micronutrients, and food groups and their related effects on gut microbiota and T2DM is an important approach to prevent and control diabetes. Although not all the effects of the interactions between food components and T2DM are completely known, it is clear that diets rich in fruits and vegetables have beneficial effects on glucose metabolism [67]. For example, foods with a low glycemic index have beneficial effects on blood sugar, HbA1c, total cholesterol, LDL cholesterol, and the inflammatory response in patients with diabetes and in the prevention of T2DM development across populations [68,69,70,71,72]. A recent study in obese women who followed a low-glycemic diet consisting mainly of whole grains, fish, vegetables, algae, and perilla oil or a control diet consisting mainly of refined rice, bread, noodles, meat, and processed foods showed a higher level of *Gemminger formicilis*, *Collinsella aerofaciens*, *Escherichia coli,* and *Bifidobacterium longum* and a lower serum butyric acid level in those receiving the control diet compared to the low-glycemic diet [73]. Gut dysbiosis and increases in abundance of pathogenic bacteria, especially *Bacteroides,* have been reported in the presence of a carbohydrate-rich diet [74]. However, not all studies showed significant differences in the glycemic control of the lipid profile in people who followed a low-glycemic-index diet compared to other types of diet [71,75,76].

Multiple studies have demonstrated associations between different dietary patterns and the risk for T2DM; however, these associations are quite complex, because people do not consume individual foods but mixtures of foods [77], causing corresponding changes in microbial composition. Further, it has been shown that long-term diets lead to the establishment of major enterotypes *Prevotella*, *Bacteroides*, and *Ruminococcus* [73,78] because diet is the main modulator of gut microbiota. *Bifidobacterium* spp., *Lactobacillus* spp., *Bacteroides* spp., *Alistipes* spp., *Bilophila* spp., *Clostridium* spp., *Roseburia* spp., *Eubacterium* spp., *Enterococcus* spp., *Faecalibacterium prausnitzii*, *Akkermansia muciniphila*, *Escherichia coli*, *Helicobacter pylori*, and *Streptococcus* spp. [79] are among the many bacteria influenced by diet. For example, the Prevotella enterotype was associated with a high intake of carbohydrates, especially sugar, while the Bacteroides enterotype was associated with a high intake of meat [73]. In addition, specific diets such as the Western Diet (WD) and the Mediterranean diet (MD) have been shown to exert differential changes in the gut microbiota composition and ensuing metabolic functions. As such, a hypercaloric diet high in fats and animal proteins, characteristic of the WD, is associated with microbial dysbiosis [80]. The WD is shown to increase unfavorable species such as *Escherichia coli* and *Ruminococcus torques* [81], which in turn promote increased gut permeability and metabolic endotoxemia through increased abundances in these Gram-negative LPS-producing bacterial genera [82], contributing to insulin resistance. At the same time, it has been shown that the WD reduces SCFA-producing bacterial genera, such as *Eubacterium* and *Roseburia* [83]. These pro-inflammatory changes in gut permeability via the WD can be attributed, to some extent, to mTOR hyperactivation, which was improved after antibiotic introduction [84]. Further, the effects of similar diets are not limited to peripheral changes but are also associated with central insulin resistance, as evidenced through the increased serine phosphorylation of IRS-1 and inflammatory responses through nuclear factor kappa beta (NFKβ) and JNK activity [85]. In addition to phosphorylating IRS-1, JNK contributes to insulin resistance through promoting metabolic inflammation and negatively regulating interactions between PPARα-FGF21 as well as contributing to adiposity through dysregulation of the thyroid-stimulating hormone (TSH) axis [86]. Studies also have shown that a WD in patients with T2DM promotes more C-peptide post-prandially, which is an endogenous marker of insulin secretion [87]. Increased insulin secretion was also reported after the consumption of a Westernized diet, which preceded peripheral insulin resistance [88].

On the other hand, the MD, characterized by a high intake of dietary fiber, nuts, whole grains, and omega-3 polyunsaturated fatty acids, has been associated with favorable effects on gut microbiota composition and hyperglycemia [89]. Interestingly, the effects on gut microbiota are largely opposite from those observed after WD adherence, with MD consumption promoting increased relative abundance in the main SCFA-producing genera like *Lactobacillus*, *Bifidobacterium*, *Eubacterium,* and *Faecalibacterium* while reducing concentrations of *Bacteroides* and *Prevotella* spp. [90,91], which collectively contribute to better glucose homeostasis. One large-scale observational study of over 22,000 human participants who adhered to the MD for 6 months showed that these individuals had a lower risk of new onset T2DM [92]. The mechanisms behind improvements in glucose homeostasis include a reduction in inflammatory processes [93], the modulation of gut hormones [94,95], and altered production of microbial metabolites [96]. For example, 12-week adherence to the MD reduced pro-inflammatory cytokine interleukin-6 (IL-6) by 49% in T2DM patients [93]. Similarly, markers of inflammation such as C-reactive protein (CRP) and intracellular adhesion molecule-1 (ICAM-1) showed significant reduction post-MD [97]. ICAM-1 is heavily intertwined in T-cell-mediated processes, indicating that the adaptive immune response is also affected by this dietary intervention [98]. In addition, the MD exerts antioxidant and anti-hyperglycemic effects by augmenting GLP-1 activity in endothelial cells. These findings are also supported by two recent human studies showing that MD adherence over 210 days or 24 weeks was associated with lower serum glucose, elevated fasting GLP-1 level, and improved insulin resistance and HgbA1c [94,95]. Importantly, some of the bacterial genera changes associated with the MD, such as decreases in *Prevotella* and *Bacteroides,* may influence markers of insulin resistance [96]. These genera have been shown to aggravate insulin resistance and cause glucose intolerance by elevating circulating levels of branched-chain amino acids (BCAAs) [96]. Studies have linked increased concentrations of BCAAs to the activation of mTORC1, a cell growth regulator, which causes the dysregulation of insulin signaling [99]. In parallel, these BCAAs are shown to be associated with pancreatic β-cell mitochondrial dysfunction and apoptosis, further contributing to insulin resistance [100]. Taken together, these findings support the role of dietary patterns in the remodeling of gut microbiota and resulting alterations in biomarkers associated with inflammation, hyperglycemia, and insulin signaling dysregulation.

## 4. Effects of Macronutrients on T2DM and Gut Microbiota

Various macronutrients exert distinct effects on gut microbiota and T2DM. In the following subsections, we explore the role of carbohydrates, dietary fibers, and starches, as well as that of proteins and lipids, in mediating these effects.

### 4.1. Carbohydrates

**Figure 2:**
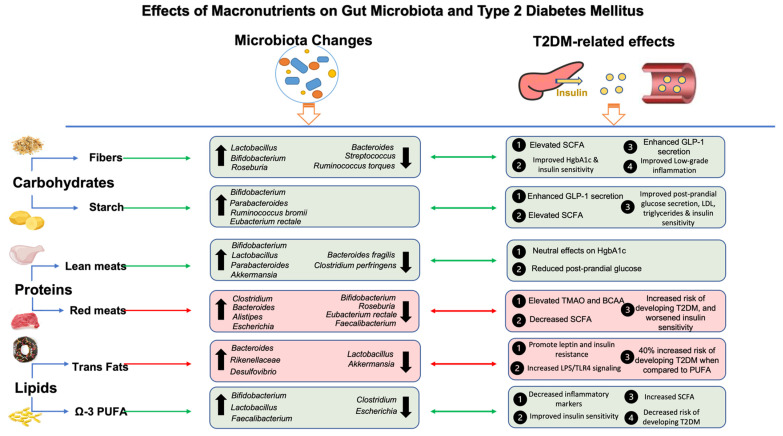
Effects of macronutrients, carbohydrates, proteins, and lipids on gut microbiota and type 2 diabetes mellitus. Carbohydrates comprise fibers and starches. Fibers and starches promote beneficial gut microbial changes, which improve insulin resistance through increased SCFA concentrations, enhancing GLP-1 secretion, improving low-grade inflammation and metabolic parameters including HgbA1c, post-prandial glucose secretion, LDL, and triglycerides. Lean meats promote beneficial gut microbial changes but have neutral effects on HgbA1c, though they do reduce post-prandial glucose secretion. Red meats generally contribute to harmful changes in microbial composition, which promote elevations in negative microbiota metabolites including TMAO and BCAAs, while decreasing SCFAs, which increases the risk of developing T2DM and worsening insulin sensitivity. Trans fats also promote harmful changes in microbial composition, which negatively affects leptin and insulin resistance, increases LPS/TLR4 binding, and is associated with up to 40% increased risk of developing T2DM when compared to polyunsaturated fatty acids. Omega-3 polyunsaturated fatty acids, on the other hand, confer beneficial effects on gut microbiota, which decreases inflammatory markers, improves insulin sensitivity, and increases SCFAs, to overall decrease the risk of developing T2DM. Abbreviations: T2DM, type 2 diabetes mellitus; SCFAs, short-chain fatty acids; HgbA1c, Hemoglobin A1c; GLP-1, Glucagon like peptide 1; LDL, low density lipoprotein; TMAO, Trimethylamine N-oxide. BCAAs, branched-chain amino acids; LPS/TLR4, Lipopolysaccharides/Toll-like receptor 4; PUFA, polyunsaturated fatty acids.

Digestible carbohydrates are enzymatically degraded in the small intestine and are represented by starch and sugars, such as glucose, fructose, sucrose, and lactose [101]. The breakdown of these compounds stimulates insulin response by releasing glucose into the bloodstream, thereby influencing insulin signaling [102]. Over the years, the relationship between carbohydrate intake, diabetes, and gut microbiota has been studied, with differences shown in the post-prandial glycemic response determined both by the amount as well as by the type of carbohydrate consumed [103,104]. Low-carbohydrate diets (LCD) have long been part of the main nutritional therapy regimen in the management of type 2 diabetes [105]. For example, an LCD, characterized by under 40% of the total energy intake being carbohydrates, had a beneficial effect on HgbA1c as compared to both very low carbohydrate content or moderate carbohydrate content (40–64% of total energy intake) [106]. ADA guidelines include the importance of diets with a low carbohydrate content in reducing HbA1c levels [107]. Numerous studies have supported the hypoglycemic effect of an LCD through decreased blood sugar and increased insulin sensitivity, leading to lowering oral antidiabetic medications, while also improving lipid parameters such as increased HDL cholesterol and decreased triglycerides [108,109,110,111]. Adherence to an LCD reduced the risk for T2DM in children and adolescents [112], while a diet high in carbohydrates increased the risk of T2DM [113], clearly demonstrating the importance of this macronutrient in glucose homeostasis. Further an LCD is shown to modulate gut hormones such as GLP-1, while concomitantly promoting beneficial changes in gut microbiota and diabetic markers [22,114]. For example, LCD consumption for three months was associated with enhanced GLP-1 secretion in humans, reduced HgbA1c, and an increased abundance of SCFA-producing species, *Roseburia*, *Ruminococcus,* and *Eubacterium* [22]. These SCFAs resulting from colonic carbohydrate fermentation act on free fatty acid receptors, FFAR2 (GPR43) and FFAR3 (GPR41), to stimulate GLP-1 release through the mitogen-activated protein kinase (MAPK)/extracellular regulated protein kinase (ERK) pathway [115]. For example, acetate increased GLP-1 secretion up to three-fold and butyrate by two-fold [115,116] in response to the administration of *Bifidobacterium*, *Lactobacillus,* and *Enterococcus* spp. Therefore, these metabolic signaling pathways involving both the host and bacteria pathways play a significant role in metabolic health in response to carbohydrate intake (Figure 2).

On the other hand, a high carbohydrate intake, including diets rich in glucose and fructose, promotes both metabolic disorders and intestinal dysbiosis [117] and has been associated with an increase in the abundance of pathogenic bacteria, especially *Bacteroides* [74]. Additionally, metagenomic sequencing data of gut microbiota in animal models consuming carbohydrate-dense diets have shown increases in the Firmicutes-to-Bacteroidetes ratio as well as in pro-inflammatory *Desulfovibrio vulgaris* and mucin-degrading *Akkermansia muciniphila* [118]. These findings were accompanied by increased glucose intolerance, elevated serum glucose and a two-and-a-half-fold increase in gut permeability [118,119]. The decrease in gut permeability may be attributed to the role of *Akkermansia muciniphila*, *Bacteroides*, and *Desulfovibrio* spp. in inflammatory processes. In general, healthy amounts of *Akkermansia* have favorable effects on gut barrier integrity and inflammation [120]; however, when they are in excess, an opposite effect is shown, with *Akkermansia* over-degrading the mucin layer leading to increased gut permeability and the secretion of inflammatory cytokines [121]. *Desulfovibrio*, a Gram-negative bacterium genera, known to produce hydrogen sulfide gas, increases T cell activity and systemic inflammation, known to cause important sequelae in both cognition and metabolic syndrome [122]. *Desulfovibrio* is also positively correlated with increases in fasting insulin, which can lead to insulin resistance [123]. Similarly, *Bacteroides* have been shown to degrade the mucin layer when abundant and exhibit virulence factors that fuel their growth in conditions that cause low bacteria diversity [124]. Importantly, *Bacteroides* spp. increase the biosynthesis of BCAAs, an important marker for increased insulin resistance [96]. Taken together, these changes caused by high carbohydrate intake can contribute to metabolic endotoxemia, insulin resistance, and hyperglycemia (Figure 2).

#### 4.1.1. Dietary Fibers, Gut Microbiota, and T2DM

Dietary fibers are plant components that are characterized by resistance to digestion and absorption in the small intestine [125]. Unlike digestible carbohydrates, dietary fiber is not enzymatically degraded in the small intestine but is fermented by microorganisms resident in the large intestine [126]. Fibers are classified according to their physico-chemical characteristics, such as fermentability, solubility, and viscosity [127]. The high intake of dietary fiber supports gut health [127] and promotes glycemic control, with recommendations of 25–50 g/day of dietary fibers in diabetic patients [128]. There is compelling evidence demonstrating the overall health benefits of diets rich in fiber and the daily consumption of whole grains and bran was associated with decreased mortality due to cardiovascular causes in patients with diabetes [106,129]. Moreover, patients with metabolic syndrome had a lower fiber intake than those without metabolic syndrome [130]. Similarly, high glycemic index diets and low fiber content are shown to induce metabolic syndrome in individuals with T2DM [131]. The effects of dietary fibers depend on their origin, noting that cereal fibers were more strongly associated with a decrease in the risk of diabetes, compared to fruit fibers, which had a weaker association [132]. However, dietary fibers derived from cereals and fruits have beneficial effects in controlling T2DM through improvements in inflammatory processes as measured through CRP and tumor necrosis factor alpha (TNF-α) levels [133]. Dietary fibers also stimulate increases in circulating adiponectin, which serves as a marker of insulin sensitivity [134]. Overall, a high fiber intake was associated with a lower risk of type 2 diabetes [135,136] (Figure 2).

The effects of fiber on glycemic control are also influenced by the fiber viscosity [137,138]. For example, the administration of psyllium, a soluble fiber, improved the lipid profile and glycemic control [139]. In general, soluble fiber has a stronger beneficial effect on T2DM compared to insoluble or non-viscous fiber. This may be due to the action of gut bacteria and its byproducts on the fiber substrate and their metabolic functions [140]. In addition, compared to insoluble fibers that are poorly fermentable and have an important role in increasing the rate of intestinal transit, soluble fibers are highly fermentable and are efficiently used by the gut microbiota [141]. Specifically, soluble dietary fibers have been shown to promote the diversity of gut microbiota and serve as one of the most important substrates for gut microbiota [140]. Following fermentation, different metabolites are generated, including short-chain fatty acids [142], with the highest proportion (60%) being acetate, followed by propionate (25%) and butyrate (15%). Other generated byproducts also include gases such as methane and carbon dioxide [5]. As previously mentioned, SCFAs are used as substrates for the metabolism of lipids, glucose, and cholesterol and have a significant role in maintaining tissue barrier function and regulating gene expression and immunoregulation. They also provide energy support for colonocytes and regulate homeostasis of the colon, by maintaining the integrity of the intestinal mucosa and reducing inflammation but also by promoting epithelial cell proliferation, differentiation, and water absorption [5]. For example, when compared to cellulose, an insoluble fiber, inulin, which is a soluble fiber, provided significant protection against high fat diet-induced metabolic syndrome [143]. These protective mechanisms included enterocyte proliferation, anti-microbial gene expression, and increased IL-22 expression, which improved low-grade inflammation and prevented the proliferation of unfavorable microbiota [143]. Therefore, dietary fiber represents an ideal source of carbohydrates accessible to the gut microbiota, that can be used to provide the host with energy and carbon sources [79]. Dietary fibers are also called prebiotics and they selectively stimulate the growth or activity of certain microorganisms [144]. The best-known sources of prebiotics are unrefined barley and oats, soy, and inulins, but they also include non-digestible oligosaccharides, such as fructans, polydextrose, fructooligosaccharides, galactooligosaccharides, xylooligosaccharides, and arabinooligosaccharides [145]. The high intake of dietary fiber is associated with the increase in the diversity of the gut microbiota, characterized mainly by the growth of *Bacteroidetes and Prevotella* spp. but also with the improvement in insulin resistance and the decrease in susceptibility to infections and malignant processes [146]. On the other hand, the lack of dietary fiber has contrasting effects, promoting decreased microbiota diversity [147] while also decreasing the production of butyrate, worsening insulin resistance, and increasing susceptibility to infections [148,149]. Importantly, the gut microbiota whose abundance is increased by dietary fibers ameliorate T2DM [145,150]. For example, *Bifidobacterium* spp. and other SCFA-related genera were shown to be increased while also enhancing GLP-1 secretion and improving HgbA1c levels [150], while harmful bacterial metabolites such as hydrogen sulfide and indole were reduced. Further, a positive correlation was observed between an increased amount of *Roseburia*, *Lachnospira,* and *Prevotella* and an increased level of short-chain fatty acids with a high intake of dietary fiber, with negative correlations with *Ruminococcus* and *Streptococcus* [151]. Similarly, a direct association between dietary fiber intake and gut microbiota diversity has also been observed in overweight pregnant women in which dietary fiber intake also decreased the abundance of *Bacteroides* [152]. These findings are consistent with other studies showing that date consumption, which contains high amounts of dietary fibers, increases the abundance of *Bifidobacterium*, while having the opposite effect on *Bacteroides* spp. [153]. At the phylum level, the overall abundance of Bacteroidetes is increased, thereby improving the Firmicutes-to-Bacteroidetes ratio [146]. Taken together, there is strong evidence for the role of dietary fibers in promoting the beneficial effects of gut microbiota and T2DM.

#### 4.1.2. Starch, Gut Microbiota, and T2DM

Starch provides approximately 20 to 40% of the energy requirements for most people and is classified according to the degree of enzymatic hydrolysis [154]. Some rapid digestible starches are hydrolyzed in less than 20 min of enzymatic digestion [154], while slow digestible starches are absorbed in the small intestine after approximately 100 min of enzymatic digestion. The resistant starch is not hydrolyzed even after 120 min of enzymatic incubation [155]. Resistant starches are considered dietary fiber found both naturally in cereals, fruits, and vegetables but also may be added into processed foods [156]. Certain resistant starches are considered prebiotics as well, with positive effects in the prevention or even improvement of metabolic diseases, including metabolic syndrome and T2DM [157]. There are currently five types of known resistant starch: type 1 resistant starch, found in whole grain or coarsely ground bread and durum wheat pasta; type 2, found in negated potato starch, green banana starch, gingko starch, and corn starch; type 3, amylose and retrograded starch; type 4, a chemically modified starch; and type 5, an amylose–lipid complex [157]. Foods such as potatoes, rice, pasta, and breakfast cereals contain less than 2.5% resistant starch. On the other hand, certain foods such as boiled legumes and peas, but also other cooked and cooled starchy foods, contain a higher amount of resistant starch (5–15%) [157]. The Western Diet contains mostly foods with a low content of resistant starch.

A growing number of studies suggest the importance of resistant starch in reducing the risk of type 2 diabetes [158,159,160]. Reductions in post-prandial blood glucose have been observed when carbohydrates from a meal were replaced with resistant starch [161]. Furthermore, the results of a meta-analysis reported that resistant starch supplementation is associated with an improvement in blood glucose, insulinemia, insulin sensitivity, and resistance, especially in patients with diabetes and overweight or obesity [160]. More specifically, type 1 and type 2 resistant starches have been associated with improved post-prandial blood glucose, and in addition, type 2 resistant starch has been associated with improved post-prandial insulin response and fasting blood glucose [162]. The use of type 3 resistant starch for 3 to 11 weeks resulted in a reduction in fasting blood glucose, triglycerides, and total cholesterol in a diabetic mice model [163]. Similarly, type 4 resistant starch introduction significantly reduced post-prandial glucose by 33% [164]. Importantly, the glycemic response following starch intake varied, with lower glycemic and insulinemia responses after raw starch intake, compared to cooked starch [165]. 

Furthermore, it is well documented that resistant starch, similar to dietary fibers, is also important in supporting the gut microbiota, through fermentation reactions mediated by resident bacteria which may explain its effects on improving insulin resistance, reduced glucose absorption, and glucose homeostasis [166]. For example, consumption of resistant starch type 4 led to an increase in the abundance of Actinobacteria and Bacteroidetes and a decrease in the abundance of Firmicutes [167]. Further, the consumption of type 4 resistant starch increased *Bifidobacterium adolescentis* and *Parabacteroides distasonis*, while type 2 resistant starch led to an increase in the abundance of *Ruminococcus bromii* and *Eubacterium rectale* [167]. Similarly, a study carried out in overweight men showed an increase in the abundance of the same species, *Ruminococcus bromii* and *Eubacterium rectale*, in men who consumed diets high in resistant starch [168]. Overall, the data support the exceptional ability of *Ruminococcus bromii* to degrade resistant starch, due to its carbohydrate active enzyme activity, and in turn, starch serves as a nutrient to increase its abundance [169,170]. These gut microbial changes caused by the resistant starch along with increases in *Akkermansia* were associated with concomitant benefits in metabolic parameters, including decreased LDL, increased GLP-1 secretion, acetate, and early phase insulin secretion [171].

### 4.2. Proteins, Gut Microbiota, and T2DM

The major functional and structural component of body cells is protein [172]. The current recommendation for protein intake for healthy individuals is 0.8 g/kg body weight per day or 10–35% of the total energy intake [173] with no difference for patients with diabetes. However, in diabetes complications such as diabetic nephropathy, the recommendation is to reduce protein intake [172,174]. Dietary proteins have received considerable attention for their role in the control of body weight given their demonstrated effects on enhanced satiety and maintenance of lean body mass during weight loss. However, the exact role of proteins in the control of diabetes is not as well defined as it is for other macronutrients [104]. Notwithstanding, an increase in protein intake has been shown to improve insulin sensitivity by maintaining muscle mass during weight loss in elderly patients with prediabetes or type 2 diabetes [173]. Likewise, a 30% calorie protein diet was associated with an improvement in some cardiovascular risk factors, though HgbA1c levels were not affected [106]. However, an improvement in insulin sensitivity was observed in a group of obese women who followed a hypocaloric and high protein diet compared to those who followed a hypocaloric and hyperglycemic diet [175], although no changes in blood glucose were observed. Further, an association has been observed between an improvement in insulin release and the maintenance of low blood sugar and milk proteins, casein, and whey [173]. Moreover, whey proteins are potent stimuli of insulin and incretin secretion such as GLP-1 and GIP that are known to lower blood sugar through the stimulation of insulin and inhibition of glucagon secretion, resulting in the inhibition of hepatic glucose production, as well as the inhibition of gastric emptying [176,177]. However, other studies did not find a positive association between increased protein intake and a lower risk of type 2 diabetes, compared to a low protein intake [113]; therefore, there is a need for more studies to examine the influence of protein on T2DM.

It is important to note that different types of proteins have differing effects on insulin signaling. For example, animal proteins promote insulin resistance and are associated with increased risk for diabetes [178,179,180,181]. On the other hand, plant proteins promote insulin sensitivity [182] and improved glycemic control in patients with type 2 diabetes [183]. Also, increasing the intake of vegetable proteins was associated with a lower probability of relapse of type 2 diabetes [184], and a lower probability of developing type 2 diabetes and its comorbidities [185]. Further, vegetable proteins are the main components of the beneficial Mediterranean diet, while animal proteins, such as red and processed meats, are characteristic of the Western Diet, which have opposite effects on both gut microbiota and T2DM as described in earlier sections. Therefore, there is strong evidence showing the importance of the type of proteins as it relates to the derangement of metabolic parameters.

Similar to other macronutrients, proteins are metabolized by gut microbes into metabolites such as short-chain fatty acids but also neurotransmitters, amino acid substrates, and organic acids that have physiological effects both locally and systemically [186]. Amino acids are fermented by gut bacteria in the distal colon, and protein fermentation leads to the lower production of short-chain fatty acids (SCFAs) and greater production of branched-chain amino acids (BCAAs) and potentially toxic substrates, such as ammonia, when compared to carbohydrate fermentation [5]. BCAAs include leucine, isoleucine, and valine, and they are not naturally synthesized in humans, making them nutritionally essential, with their most common food source being proteins. Their role in insulin resistance is thought to be threefold via the activation of mTORc1 uncoupling of IRS-1, mitochondrial dysfunction through toxic accumulation of BCAAs, and altered expression of genes in humans (BCKDHA, PPM1K, IVD, and KLF15) contributing to altered insulin signaling and therefore resistance [100]. Importantly, it has been well documented that animal proteins are metabolized into a higher content of BCAAs than plant proteins, which can explain the differences in insulin resistance between the two protein types [187]. In turn, dietary proteins contribute to changes in microbial composition. For example, a high protein diet was associated with a reduction in the abundance of propionate- and butyrate-producing bacteria such as *Akkermansia*, *Faecalibacterium*, *Roseburia*, and *Eubacterium* while increasing the abundance of *Escherichia*, *Shigella*, *Enterococcus*, and *Streptococcus* [188,189] in a rodent model. These findings are consistent with data showing a decrease in fecal butyrate but not propionate or acetate in response to high protein intake in humans [190]; but, see also [191], where no significant results were seen, although the study populations differed between the studies (overweight vs. endurance athletes).

Additionally, an increased abundance of *Clostridium*, unnamed *Clostridiales*, and *Allobaculum* and decreased relative concentrations of Eubacterium, *Akkermansia*, *Mucispirillum*, *Ruminococcus*, *Johnsonella*, *Alistipes*, *Butyrivibrio*, and *Blautia* were also observed after high protein intake [192]. A similar increase in the abundance of *Bacteroidaceae* was observed with a high protein intake, given that nitrogen from dietary proteins promotes an increase in *Bacteroidaceae* [193]. Increased nitrogen production from gut microbiota and resulting reactive nitrogen species generally relates to the growth of unfavorable microbial genera, as SCFAs, particularly butyrate, have been shown to limit its production [194]. Further, a high-protein hypoglycemic diet decreased amounts of *Roseburia* and *Eubacterium rectale,* which correlated with a decrease in fecal butyrate [195]. In addition to decreased SCFAs, there was an increase in trimethylamine N-oxide (TMAO), a bacterial byproduct with proatherogenic and pro-diabetic effects, that is positively correlated with increased concentrations of anaerobic bacteria such as *Bacteroides*, *Alistipes,* and *Bilophila* as well as animal products, including red meat sources of protein [79,196]. An increase in the abundance of *Bacteroides* and *Clostridia* and a decrease in the abundance of *Bifidobacterium adolescentis* have also been seen in individuals consuming a diet rich in beef compared to those who did not consume meat [197]. Similar changes in the microbiota composition following protein consumption have been reported at the phylum and class levels. For example, a high abundance of Firmicutes, especially Clostridia and Bacilli, was observed in rats fed beef, pork, or fish protein. An increase in the abundance of Bacteroidetes was seen in rats fed soy protein, while a decrease in Bacteroidetes’ abundance was noticed in rats fed fish protein [198]. Compared to animal proteins, plant proteins increase *Bifidobacterium* and *Lactobacillus* but also decrease the amount of *Bacteroides fragilis* and *Clostridium perfringens* [5]. For example, soy proteins have been associated with promoting *Bifidobacterium*, *Lactobacillus*, *Butyricicoccus*, *Parabacteroides*, *Lachnospiraceae,* and *Akkermansia muciniphila* [199]. Collectively, these studies demonstrate the intricate relationship between protein intake, changes in the gut microbiota composition, and how these taxonomical shifts may influence hyperglycemia- and T2DM-related parameters.

### 4.3. Lipids, Gut Microbiota, and T2DM

Lipids are considered naturally occurring compounds composed of fatty acids or related derivates that are soluble in organic solvents but insoluble in water [200]. Lipid intake has somewhat controversial direct effects on blood glucose [104], though it may influence insulin sensitivity [201]. The current nutritional recommendations are to decrease the consumption of saturated lipids and trans fatty acids and increase monounsaturated and polyunsaturated fatty acids [202]. It has been consistently shown that diets high in fats increase the risk of type 2 diabetes through impaired glucose tolerance and the binding of insulin to its receptors, resulting in altered glucose transport and the accumulation of triglycerides in skeletal muscles [203]. In contrast, a hypolipidemic diet had no effect on the incidence of diabetes after 8 years, compared to a control diet [204], even when compared to low-carbohydrate diets [205]. Therefore, the type and quality of lipids consumed are more important in the risk of developing T2DM [203]. 

Similar to proteins, lipids from plant sources provide better health benefits than lipids from animal sources [206], and a high intake of plant-based lipids has been associated with a significantly lower risk of the occurrence of T2DM [113]. Results from a meta-analysis report an inverse correlation between the incidence of T2DM and the high intake of vegetable-based lipids, especially plant-derived α-linolenic acid and polyunsaturated fatty acids [202]. Conversely, the intake of trans fatty acids has been associated with all-cause mortality, T2DM, and ischemic heart disease [207]. Similarly, saturated fats cause unfavorable changes in energy balance, insulin resistance, and fat-cell differentiation [208], though all-cause mortality was not shown to be increased the same way it was after trans fat consumption [207]. Interestingly, replacing dietary saturated fat with omega-6 polyunsaturated fatty acids resulted in a lower risk of diabetes and related sequelae [209]. A higher intake of omega-6 polyunsaturated fatty acids was associated with a lower risk of diabetes [210]. More specifically, replacing saturated fatty acids with polyunsaturated fatty acids (PUFAs) was associated with a 35% decrease in the risk of developing T2DM, and replacing trans fatty acids with PUFAs correlated with a 40% reduction in the same parameter [211]. Therefore, the most beneficial lipids in promoting better insulin sensitivity are PUFAs, with omega-6 and omega-3 PUFAs being extensively studied [212]. A recent meta-analysis of 67 studies showed that increased supplementation of omega-3 PUFA decreased the risk of developing T2DM [212]. Importantly, the mechanisms of the beneficial effects of PUFAs involves gut microbiota and related decreases in production of inflammatory mediators [213]. Specifically, studies have demonstrated increased *Bifidobacterium*, Bacteroidetes-to-Firmicutes ratio, and fecal SCFAs, concomitantly with the attenuation of high fat diet-induced insulin resistance and liver inflammation, following omega-3 PUFA introduction [214]. Inflammatory markers such as IL-1β, TNF-α, IL-8, IL-6, and interferon-γ were significantly reduced following omega-3 supplementation, which also improved fat accumulation and metabolic parameters [215].

Conversely, the increased consumption of dietary lipids, particularly saturated fatty acids and trans fats, influences the composition of the gut microbiota negatively, decreasing bacterial diversity [216] and increasing the Firmicutes-to-Bacteroidetes ratio [216,217]. These effects also contribute to the development of obesity through leptin resistance and the promotion of low-grade systemic inflammation through the LPS/TLR4 pathway, that are characteristics of dysbiosis [218,219]. A decrease in bacterial diversity and an increase in the abundance of *Faecalibacterium prausnitzii* was also observed following the increased consumption of saturated fats [152,220,221]. Also, an increased intake of fats is also associated with an increase in the abundance of *Rikenellaceae* and *Bacteroides* and other anaerobic genera [79,222]. Similarly, a high intake of trans fatty acids was associated with a decrease in the abundance of Bacteroidetes and an increase in the abundance of Proteobacteria and *Desulfovibrionaceae* [223]. The opposite is also true, with the low intake of these unfavorable lipids resulting in relatively increased abundance of beneficial bacteria such as *Bifidobacterium* but also with improvements in glycemia and total cholesterol [220]. For example, an increase in the abundance of *Bifidobacterium*, *Adlercreutzia*, *Lactobacillus*, *Streptococcus*, and *Akkermansia muciniphila* was shown in mice given fish oil and an increase in the abundance of *Bacteroides* and *Bilophila* was shown in mice given lard, which aggravates white adipose tissue inflammation [224]. Overall, dietary lipids serve as an important macronutrient in modulating gut microbiota composition and metabolic parameters underlying T2DM.

## 5. Effects of Micronutrients on T2DM and Gut Microbiota

Micronutrients, including vitamins and minerals, have also been heavily implicated in gut microbial remodeling and glucose homeostasis. In the following subsections, we describe the role of various vitamins and minerals in modulating these processes.

### 5.1. Vitamins, Gut Microbiota, and T2DM

Vitamins are organic substances classified as either fat-soluble (vitamin A, D, E, and K) or water-soluble (vitamin B complex and C) with antioxidant vitamins such as A, C, D, and E shown to be decreased in individuals with diabetes, while vitamin D deficiency is associated with the development of diabetes and its sequelae [225,226]. For example, 6-month vitamin D supplementation improved HgbA1c, with a decreased production of oxidation products and oxygen free radicals [227]. Similarly, high-dose cholecalciferol, the active form of vitamin D, improved clinical manifestations of cutaneous microcirculation, inflammatory markers, and peripheral neuropathy [228]. Supplementation also decreases inflammatory gene expression, particularly of IL-6, IL-10, and IFN-γ, which serves as a potential benefit in protecting against T2DM development and disease progression through their roles in influencing platelet-mediated inflammation [229,230]. Further, cholecalciferol decreases insulin resistance through the increased activity of insulin receptors and enhanced expression of PPAR-γ [231]. This is also demonstrated in individuals with obesity or overweight where supplementation reduced fasting blood glucose, decreased truncal subcutaneous fat, and reversed to normoglycemia [232]. Gut microbiota, vitamin D, and the development of metabolic diseases, including T2DM [233], has been described as a three-way axis with vitamin D anti-inflammatory actions playing a central role. Particularly, vitamin D binding to the vitamin D receptor (VDR) influences gut microbial composition, with findings showing increased *Bifidobacterium* and *Akkermansia* species, which exert anti-inflammatory effects and improve insulin resistance [234]. The VDR can degrade lithocholic acid, a secondary bile acid, as well as regulate enzymes that mediate cholesterol’s conversion into bile acids [235]. Interestingly, animal studies have shown that vitamin D upregulates tight junction proteins to improve intestinal barrier integrity, reduce LPS production, and suppress hyperinsulinemia, hyperglycemia, and neuroinflammation [236]. Vitamin A supplementation has also been shown to exert beneficial effects in modulating microbiota, enhancing pancreatic β-cell activity and reducing inflammatory responses [237], though the literature is not as robust as compared to that on vitamin D. 

**Figure 3:**
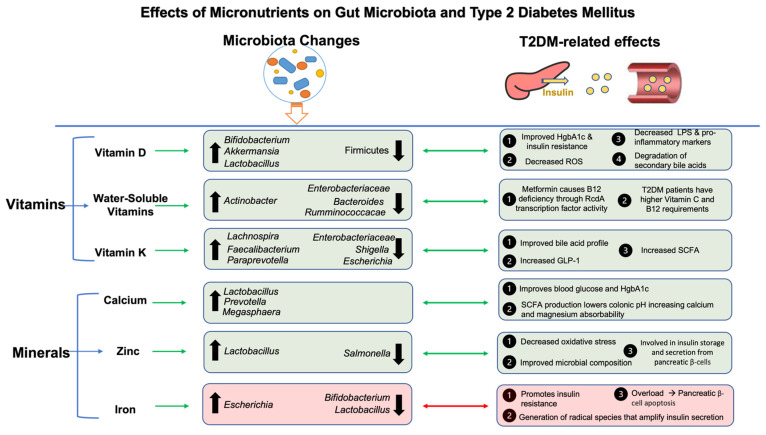
Effects of micronutrients on gut microbiota and type 2 diabetes mellitus. Vitamins generally promote beneficial changes in gut microbiota. Vitamin D has been shown to promote beneficial effects on HgbA1c and insulin resistance through decreasing reactive oxygen species, pro-inflammatory markers, and the degradation of excess secondary bile acids. Water-soluble vitamins including B12 are affected by metformin, the first-line treatment for T2DM, while vitamin C and B12 requirements are increased; therefore, supplementation is important in patients with the condition. Vitamin K improves the bile acid profile, increases GLP-1, and increases SCFAs to improve metabolic parameters. Minerals including calcium and zinc have beneficial effects on microbiota, while iron excess is associated with more negative changes in bacterial composition. Calcium improves blood glucose and HgbA1c, while increasing the relative abundances of SCFAs to increase mineral absorption through lowering colonic pH. Zinc decreases oxidative stress and is involved in insulin storage and secretion in pancreatic β cells. Iron, on the other hand, increases inflammatory species’ abundance, such as *Escherichia*, to promote insulin resistance, radical oxygen and nitrogen species, and β-cell apoptosis. Abbreviations: T2DM, type 2 diabetes mellitus; SCFAs, short-chain fatty acids; HgbA1c, Hemoglobin A1c; GLP-1, Glucagon like peptide 1; LPS, lipopolysaccharides; ROS, reactive oxygen species.

Vitamin K is another fat-soluble vitamin whose metabolism has been intricately related with gut microbiota, with recent meta-analyses demonstrating its benefits in T2DM risk [238], specifically through the improvement in fasting blood glucose and insulin resistance scores. Recent studies have shown the importance of vitamin K2 supplementation in improving glycemic homeostasis and insulin sensitivity in T2DM via gut microbiota [239]. Specifically, after 6 months of vitamin K2 supplementation, there were significant reductions in fasting serum glucose, insulin, and HbA1c levels in patients with T2DM and significant glucose tolerance improvement in diet-induced obesity mice. In addition, increased concentrations of secondary bile acids (lithocholic and taurodeoxycholic acid) and short-chain fatty acids (acetic acid, butyric acid, and valeric acid) were found in human and mouse feces that were accompanied by an increased abundance of the genera responsible for the biosynthesis of these metabolites. The further fecal microbiota transplant of these microbiota into a high fat diet-induced obesity rat model showed significant improvements in glucose tolerance through the activation of colonic bile acid receptors, increased GLP-1, and anti-inflammatory effects [239] (Figure 3). 

Water-soluble vitamins have also been implicated in various facets related to T2DM [240]. For example, patients with prediabetes and T2DM have a higher vitamin C [240] requirement than those without; thus, dietary approaches may help with the treatment of the condition. Further, treatment with metformin, currently the first-line treatment of T2DM, has been shown to cause vitamin B12 deficiency [241]. A recent study showed that metformin can assist gut microbiota in contributing to this deficiency [242]. Using functional and genomic analyses via high-throughput screens for E. coli and C. elegans, it was shown that metformin helps bacteria gather B12 from the environment by increasing the expressions of B12 transporter genes, thus reducing the B12 supply to T2D patients taking the drug over time [242]. It is important to note that vitamin B12 deficiency can further exacerbate the sequelae of T2DM such as peripheral neuropathy and even macrovascular complications in patients with the condition [243]. Therefore, B12 supplementation is important in patients on metformin treatment. Collectively, there is strong evidence showing the impact of vitamins on gut microbiota and associated T2DM metabolic abnormalities (Figure 3). 

### 5.2. Minerals, Gut Microbiota, and T2DM

Minerals, including zinc, calcium, selenium, potassium, magnesium, copper, and iron, are found in various food groups that serve an essential role in health, as well as glucose metabolism [244]. For example, zinc has been shown to have important effects in activating the cell signaling pathway that can prolong the action of insulin and modulate insulin receptors [245]. Specifically, zinc is highly involved in insulin processing, storage, and secretion in pancreatic β cells, with two zinc molecules required to coordinate these processes [246]. Further, zinc serves as an important antioxidant that improves markers of diabetes mellitus through the reduction in reactive oxygen species (ROS) [247]. Some of these processes may also be mediated by gut microbiota, as zinc deficiency has been shown to promote unfavorable effects on microbial composition and inflammatory markers [248]. Commensal bacterial species work to increase the bioavailability of zinc and iron, while pathogenic species promote the opposite [249]; therefore, dysbiosis in T2DM that increases unfavorable gut microbial composition may, to a certain extent, be attributed to zinc and iron deficiency [250]. 

Iron is the most abundant trace metal in the human body and has also been intricately related to glucose metabolism [251]. Pancreatic β cells are heavily involved in controlling iron homeostasis through the release of hepcidin, which binds transferrin, the molecule that transports iron in the blood [252]. Further, iron deficiency is correlated with impaired insulin release [253]. The Fenton reaction, which generates radical species from iron, can amplify glucose-induced insulin secretion [254]; however, when ROS accumulate in states of iron overload, these redox reactions can lead to insulin resistance and pancreatic β cells’ apoptosis [255]. As such, iron supplementation has been shown to have negative effects on gut microbial composition, with observed decreases in *Lactobacillus* and *Bifidobacterium*, and with relative increases in pro-inflammatory *Escherichia coli* [256]. Therefore, low-iron diets can protect against the development of metabolic disease through improved TGF-β signaling [257]. Similarly, trace elements such as copper, when in excess, have similar negative effects through the production of reactive oxygen species, promoting insulin resistance [245], though the effects of copper on gut microbiota are not well understood.

Calcium is the most abundant mineral in humans with calcium signaling influencing insulin secretion and resistance through its systemic importance and associations with other vitamin and mineral deficiencies such as hypomagnesia and hypovitaminosis D [258]. Study findings have shown that hypocalcemia and hypomagnesia are correlated with increased plasma blood glucose and HgbA1c in humans [259]. A favorable gut microbiome is essential for optimal calcium absorption, with SCFA production found to lower the pH in the colon, making calcium more soluble and therefore absorbable [244]. The interrelationship between gut microbiota and magnesium is similar, with magnesium supplementation promoting *Lactobacillus* spp. Production, and reciprocally, the resulting SCFA production can improve mineral absorption (Figure 3). Therefore, states of dysbiosis, such as T2DM, contribute to mineral deficiencies by impairing mineral metabolism, absorption, and other important processes [260].

## 6. Food Groups and Related Effects on Gut Microbiota and T2DM

Considering the effects of macro- and micronutrients on gut microbiota and T2DM discussed above, food groups and dietary patterns are major determinants of the gut microbiota–metabolic disorder axis. Therefore, in the following sections, we present the interrelations between food groups (cereals, whole grains, fruits, vegetables, dairy products, meat products, and oilseeds), gut microbiota, and T2DM and how food patterns and adherence to certain diets modulate the relative abundance of several gut microbiota taxa and their impact on physiologic, metabolic, and hormonal functions that impinge on the prevention, development, and management of T2DM.

### 6.1. Cereals and Cereal Products

Cereals are composed of whole grains, wheat, oats, rye, and barley, which have been shown to have beneficial effects on metabolic health and gut microbiota [261,262]. Over the years, an increasing number of studies have elucidated the effects of cereal products on T2DM, largely showing that the components within cereals decrease the risk of developing the disease and related sequelae [263,264,265,266]. Dietary recommendations for cereals in metabolic disease include increasing the intake of cereals with whole grains and limiting the intake of refined grains or cereals with processed sugars and artificial sweeteners [267]. Both whole wheat intake and the intake of barley, oat, and rye have been associated with improved blood glucose levels [268,269] and increased insulin sensitivity [270]. It should be noted that the beneficial effects of cereals appear when the intake is high, at least 4 g of β-glucans daily [269,271]. For example, a supplement of up to 50 g of whole grains per day was associated with a 25% decrease in the risk of T2DM [272]. Similarly, the consumption of two servings of whole grains per day was associated with a 21% decrease in the risk of T2DM [273], while a refined grain intake of 200–400 g per day was associated with a 6–14% increase in the risk of T2DM [272]. The composition of the whole grains such as magnesium, phytochemicals, isoflavins, and lignins was also associated with beneficial effects in T2DM [274]. Adding cereal fiber to meals reduced post-prandial insulin release, indicating the important roles of fiber in improving insulin sensitivity [266]. Taken together, these data provide strong evidence for cereal products in reducing the risk of T2DM development. 

Further, cereal-based dietary approaches are shown to affect multiple metabolic parameters in individuals already diagnosed with T2DM, some of which may be related to changes in gut microbiota. For example, after a 3-month adherence to high dietary fiber-based cereals, there were favorable trends in lipids, HgbA1, body mass index (BMI), adipose distribution, and fasting C-peptide levels [263]. Similarly, high fiber rye, a component of healthy cereal, is shown to improve similar parameters when compared to refined wheats [264]. In addition to improving metabolic parameters, the high fiber rye diet produced important changes in gut microbiota, including elevated SCFA-producing *Agathobacter* and decreased *Ruminococcus torques*, with associated increases in plasma butyrate concentrations [264]. When compared to refined grains, the whole grain has immunomodulatory effects that were associated with microbial composition alterations [275]. For example, the introduction of wheat grains after a 2-week Western-style diet improved SCFAs, increased SCFA-producing *Lachnospira*, and reduced the pro-inflammatory *Enterobacteriaceae* family which correlated with positive changes in effector memory T-cell activity and acute innate immune response [275]. Other immunomodulatory effects of cereals have also been described in the literature, with the reduced activity of pro-inflammatory cytokines, TNF-α and IL-6, being observed after consumption [276,277]. In rodent studies, the effects of wheat also improved GPR41/43 receptor expression and enhanced GLP-1 secretion with concomitant increases in SCFA-producing bacteria, providing further insights into the multitude of effects that cereals have on metabolic disease [278].

In addition to the changes described above, it seems that a general increase in *Bifidobacterium* and *Lactobacillus* spp. is common after cereal consumption, an effect consistent with other recent studies [265]. Previous studies have also shown that diets rich in whole wheat compared to refined wheats exhibit an abundance of *Bifidobacterium* and relative decreases in *Bacteroides* after a 12-week intervention [279]. The type of cereal consumed is also important in determining microbiota shifts. For example, an increase in the abundance of *Bifidobacterium* and *Lactobacillus* was seen in the gut microbiota of people who consumed whole grain cereals for breakfast, compared to the microbiota of people who consumed cereals based on wheat bran [280]. A corn-based cereal diet increased the abundance of fecal *Bifidobacteirum* after a 3-week intervention, as compared to a refined-corn-based cereal [281]. These changes in the composition of the gut microbiota could be observed even at a low intake of whole corn (29.6% of the recommended total of 48 g [281]). However, the opposite is also true, with the sugar additives and processing seen in refined cereals having been shown to have negative effects on both gut microbial composition and related metabolites [282]. Therefore, it is evident that eating cereals with naturally occurring fibers can be beneficial in preventing or treating metabolic derangements in T2DM, while avoiding refined cereals and cereals with additives is also important. 

### 6.2. Fruits and Vegetables 

In general, some of the healthiest foods are considered vegetables and fruits, due to their content of dietary fiber, vitamins, minerals, and flavonoids [283]. Multiple studies have demonstrated the inverse associations between the consumption of green leafy vegetables and the risk of developing T2DM [67,284,285], the consumption of fruits and T2DM [286], as well as the intake of mixed fruits and vegetables and T2DM [287]. Specifically, an intake of 0.2 servings per day of green leafy vegetables reduced the risk for type 2 diabetes by 13% [284], with similar findings in another meta-analysis showing a risk reduction of 14% [285]. Changes in microbial shifts after the consumption of fruits and vegetables have also been described with study findings showing a decreased abundance of the *Lachnospiraceae* family, including *Ruminococcus*, and increased concentrations of *Faecalibacterium* and *Lactobacillus* [288]. Further metagenomic sequencing studies combining two large human cohorts have shown changes that include an increased abundance of *Faecalibacterium prausnitzii*, *Akkermansia muciniphila*, *Ruminococcaceae*, *Clostridiales*, and *Acidaminococcus* and a decrease in the abundance of *Fusobacterium* [289].

Vegetables and fruits are sources of antioxidants that have been associated with augmenting glucose metabolism, by improving oxidative stress [290], particularly given their high content of flavonoids and polyphenols [291]. Interestingly, flavonoids are shown to modulate gut microbiota-related metabolic processes, particularly through the suppression of lipogenesis and the upregulation of lipolysis, via the FXR pathway in bile acid metabolism [292]. These effects of flavonoids were corelated with increased *Akkermansia* and reductions in *Lachnoclostridium*, *Desulfovibrio*, *Colidextribacter,* and *Blautia*, all of which are strongly associated with metabolic parameters [292]. Further, flavonoid-based dietary interventions alleviated inflammation as measured through LPS/TLR-4, TNF-α, IL-6, and IL-10, while also improving insulin resistance, HgbA1c, and oral glucose tolerance [293]. Interestingly, GLP-1 release was also enhanced following flavonoid introduction. The beneficial effects of fruit and vegetable flavonoids also are shown by the improvement of intestinal barrier integrity, as well as promoting islet cell proliferation and the suppression of islet cell apoptosis [294]. Flavonoids also modulate glucose metabolism by the upregulation of the IRS/AKT signaling pathway to increase GLUT4 translocation and the synthesis of glycogen, while concomitantly improving the Firmicutes-to-Bacteroidetes ratio [295]. As such, flavonoids, a major component of fruits and vegetables, exert a multitude of metabolic benefits at the intersection between gut microbiota and glucose homeostasis.

In addition to flavonoids, fruits and vegetables are comprised of other beneficial bioactive phytochemical-based nutrients, including vitamin C and carotenoids, which contribute to insulin sensitivity [296,297]. Also, green leafy vegetables contain magnesium which is inversely associated with an increased risk for type 2 diabetes [298]. The association between fruit and vegetable intake and a reduced risk of type 2 diabetes may be due to their dietary fiber content [299] and the subsequent effects of weight loss in overweight individuals [300]. Fruit and vegetable juices, depending on their content, may have differing outcomes on both gut microbiota and T2DM [301,302,303,304]. Fruit juices that are altered by added sugar or artificial sweeteners pose harmful risks to the gut metabolic profile [301]. For example, the artificial sweetening of fruit beverages results in modest changes in gut microbiota, particularly in the ratio of Firmicutes to Bacteroidetes [301]. However, the introduction of natural fruit or vegetable extracts or juice generally has favorable effects [302,303,304]. In a prediabetic rodent model, blueberry juice improved the microbiota composition as well as metabolic parameters including insulin signaling, inflammation, ketogenesis, and fatty acid oxidation [303]. Similarly, pomegranate juice can reduce the post-prandial glycemic response after eating a high-carbohydrate meal, primarily breads [305]. Overall, fruits and vegetables are an important food group in maintaining a healthy microbiota profile because diets high in fruits, vegetables, legumes, and whole grains are accompanied by optimal body weight, reduced inflammation, and lower insulin resistance.

### 6.3. Milk and Dairy Products

Dairy products are rich in protein, B vitamins, and minerals, such as calcium, magnesium, potassium, phosphorus, and zinc, all of which have important effects on gut microbiota composition [306]. Dairy proteins, especially whey proteins, are associated with improved insulin sensitivity and a reduced risk of type 2 diabetes [307]. Interestingly, high quantities of dairy consumption (two servings per day) in adolescence were associated with a 38% decreased risk of developing T2DM in middle-aged women [308]. Further, an inverse correlation was observed between the intake of skimmed or semi-skimmed dairy products and the risk of type 2 diabetes [309]. This decreased risk was seen with 200 g of skimmed dairy product intake, with an improvement in risk up to 6% with every additional 200 g, up to a daily total of 600 g [272]. Another study has shown that one serving of dairy per day has beneficial effects on T2DM risk reduction of 9% in men and 4% in women [310,311]. Dairy consumption produces specific compositional changes in gut microbiota. For example, the introduction of dairy products or intake of yogurt for three weeks led to decreased *Bacteroides fragilis* [306] and an abundant growth of *Lactobacillus* and *Bifidobacterium* [312]. Similarly, the consumption of kefir, a yogurt-based drink, over the next 4 weeks increased the abundance of *Lactobacillus* [313,314], with associated elevated levels in fecal SCFAs [314]. Interestingly, in studies on murine models, yogurt-derived *Lactobacillus plantarum* has been shown to ameliorate the reduction in pancreatic β-cell mass with notable improvements in insulin resistance [315]. Taken together, these studies show that dairy consumption prompts significant changes in the composition of gut microbiota that are beneficial to the host in mitigating the deleterious effects of T2DM.

### 6.4. Meat and Meat Products

The recommendations for patients with type 2 diabetes regarding the intake of meat and meat products are similar to the recommendations for healthy individuals, i.e., one portion/day or the equivalent of 100–150 g of lean meat per day [316]. Lean meat and meat products are sources of protein with high biological value, but they are also important sources of iron and vitamin B12 [317]. However, red meats are shown to exert negative effects in both contributing to T2DM development and worsening the condition [316]. Several positive associations have been reported between the intake of processed red meats and increased blood glucose concentrations, insulin levels, and risk for obesity [318,319]. Moreover, the risk for type 2 diabetes was associated with the intake of red meat up to 100 g per day [272] but also with the intake of up to 50 g per day of processed meat products [272,320]. These effects have been attributed to the content of heterocyclic amines and nitrates affecting glucose metabolism [321,322]. These metabolites contribute to insulin resistance through adverse effects on pancreatic β-cell function and insulin-like growth factor (IGF-1) [323]. Further, these inorganic nitrates, present in processed meats, promote DNA damage through conversion to cytotoxic agents such as peroxy-nitrite as well as reactive oxygen species, which increase pro-inflammatory cytokine production and hamper glucose homeostasis [324]. Red meats also enhance the presence of dietary advanced glycosylated end products (dAGEs), the result of the Maillard reaction that occurs between amino acids and reducing sugars [325]. These dAGE products are shown to increase insulin resistance, while a restricted intake of dietary glycoxidation products improved insulin sensitivity in diabetic mice [326]. Additionally, it has been shown that hyperglycemia further enhances the glycation process, thus worsening the complications of uncontrolled diabetes. Therefore, red meats are a source of inorganic nitrates and substrates for the generation of dAGEs, which may contribute to the development of insulin resistance and complicate pre-existing diabetes.

Red meat may also be detrimental to gut microbial composition. It has been shown that red meat decreases *Lactobacillus*, *Paralactobacillus*, and *Prevotella*, while also decreasing SCFAs in animal models [327]. Further, the administration of beef, a red meat derivate, in mouse and rat study models led to an increase in the amount of *Clostridium* and *Blautia* and a decrease in the amount of *Bifidobacterium* and *Akkermansia* [328]. The addition of butyrate containing starch was shown to reverse the negative effects of red meat diet adherence through increased abundances of *Clostridium coccoides*, *Clostridium leptum*, *Lactobacillus* spp., *Parabacteroides distasonis*, and *Ruminococcus bromii*, but it also showed a decrease in the amount of *Ruminococcus torques*, *Ruminococcus gnavus*, and *Escherichia coli* [329]. However, the effects on gut microbial composition are dependent on the type of meat and proteins they contain [330]. For example, a study evaluating the gut microbiota of individuals consuming chicken meat is characterized by the highest proportion of Prevotella 9 (22.45%), followed by *Dialister*, *Faecalibacterium*, *Megamonas*, *Prevotella*, *Roseburia*, *Alloprevotella*, *Ruminococcaceae*, *Eubacterium,* and *Succinivibrio*, while the gut microbiota of individuals consuming pork is characterized by the highest proportion of *Bacteroides* (17.3%), followed by *Faecalibacterium*, *Roseburia*, *Dialister*, *Ruminococcus*, *Blautia*, *Megamonas*, *Agathobacter*, *Subdoligranulum,* and *Eubacterium* [331]. On the other hand, pork intake decreased the amount of *Blautia*, *Bifidobacterium,* and *Alistipes* and increased the amount of *Akkermansia muciniphila* and *Ruminococcaceae* [332]. Collectively, the intake of pork meat induced low-grade inflammation and induced oxidative stress and the upregulation of lipid metabolism genes such as PPAR-α and PPAR-γ [332]. Further, an increase in the abundance of *Lactobacillus* and a decrease in SCFA levels and SCFA-producing bacterial species such as *Fusobacterium*, *Bacteroides,* and *Prevotella* have been reported in laboratory mice fed beef, pork, or fish proteins, compared to mice that were given protein from sources other than meat, such as soy or casein [333]. Similarly, laboratory rats fed chicken meat had the highest abundance of *Lactobacillus*, compared to laboratory rats fed soy, which had the highest abundance of *Ruminococcus* and the lowest abundance of *Lactobacillus* [334]. The results of a systematic review showed that the administration of beef in mouse and rat study models led to an increase in the amount of *Clostridium* and *Blautia* and a decrease in the amount of *Bifidobacterium* and *Akkermansia* [328]. Collectively, these changes indicate that meats derived from chicken have more favorable effects on gut microbiota and insulin resistance as compared to pork and red meats.

### 6.5. Nuts, Oils, and Oilseeds

Tree nuts have been shown to exert favorable effects on gut microbiota and metabolic parameters [335,336]. For example, replacing starchy foods with peanuts or almonds in patients with type 2 diabetes led to improvements in blood glucose, HgbA1c, and inflammatory markers [335]. In addition, the daily intake of raw or roasted almonds for 4 weeks promoted *Bifidobacterium* spp. and *Lactobacillus* spp. and inhibited the growth of *Enterococcus* spp. Interestingly, the administration of raw almonds had a greater *Bifidobacteria*-promoting effect than roasted almonds, with both roasted and raw almonds having a potential prebiotic effect, including regulating gut bacteria and improving metabolic activities [336]. Similarly, nut intake promotes an increase in the abundance of *Faecalibacterium*, *Clostridium*, *Dialister,* and *Roseburia* and a decrease in the abundance of *Ruminococcus*, *Dorea*, *Oscillopira,* and *Bifidobacterium* [337]. Pistachio consumption led to an increase in the abundance of potentially beneficial, butyrate-producing bacteria [338], while eating whole, roasted, or chopped almonds is associated with an increase in the abundance of *Lachnospira* and *Roseburia* [339]. These alterations in gut microbiota were associated with a concomitant decrease in pro-inflammatory secondary bile acid production and LDL cholesterol, two interrelated parameters in the development of hyperglycemia and insulin resistance [337]. Also, a diet enriched with 20% peanut protein was effective in increasing the amount of *Bifidobacterium* and reducing the amount of *Enterobacteria* and *Clostridium* perfringens in rats [340].

Oilseeds are important sources of polyunsaturated and monounsaturated fatty acids [341,342] and their consumption has been associated with a decreased risk for type 2 diabetes [343]. For example, dietary flaxseed oil, given its rich composition of omega-3, was associated with decreased Firmicutes and pro-inflammatory markers such as IL-1β, TNF-α, and IL-6 and increased Bacteroidetes and *Alistipes* that negatively correlate with LPS production [344]. Further, the direct markers of hyperglycemia showed significant improvement, particularly in fasting blood glucose and glycated hemoglobin. Interestingly, superoxide dismutase (SOD) activity was increased as well, with previous studies showing that SOD activity can improve diabetes-induced mitochondrial electron transport dysfunction and diabetes complications such as retinopathy [345]. Meta-analyses of human studies confirm these beneficial anti-inflammatory effects of oilseeds, with decreased CRP and IL-6 activity leading to improved endothelial function and metabolic activity [346]. Oilseeds cause significant changes in the gut composition profile, such as increased *Lactobacillus* spp. and SCFAs, with reduced production of harmful metabolites such as TMAO [347]. As such, oilseeds and nuts serve as healthy food sources that are intricate components of the Mediterranean diet and modulate important metabolic processes associated with T2DM. A summary of food groups and the mechanisms by which they impact T2DM is presented in Table 1.

## 7. Effects of Nutrition on Gut Microbiota in Individuals with Comorbid T2DM and COVID-19

Given the intricate and dynamic relationship between gut microbiota and the host, gut microbiota play a critical role in individuals with comorbid COVID-19 and T2DM. For example, COVID-19 infection is shown to exacerbate microbiota-related alterations in gut microbes, including relative increases in *Enterobacteriaceae* and fungal species belonging to genera *Candida* and *Aspergillus* in patients with T2DM [348]. At the same time, butyrate production was diminished, and associated genera were reduced, with the overall alterations being associated with worsening inflammatory markers [348]. Furthermore, the microbiome analysis of patients with comorbid COVID-19 and T2DM showed significant correlations between the overgrowth of pathogenic species, with a concurrent decrease in normal gut flora, when compared to those with COVID-19 without T2DM [349]. The introduction of probiotics through dietary supplementation prevented the pathogenic bacterial overgrowth described in patients with comorbid COVID-19 and T2DM [349].

Importantly, microbiota might influence the disease severity of COVID-19 in the setting of T2DM through the production of important metabolites, such as SCFAs and TMAO [350]. As mentioned above, butyrate and other SCFA-producing bacterial spp. including *Lactobacillus* and *Bifidobacterium* are decreased in individuals with comorbid COVID-19 and T2DM, with concurrent elevations in pathogenic *Clostridium* spp. [348,351]. As such, the significant decline in SCFA production diminishes the overall immunomodulatory benefits, thereby exaggerating the immune response to COVID-19 [352], an effect that is worsened in populations with underlying T2DM and related dysbiosis [353]. In addition to immunomodulatory effects, ACE2 receptors play an essential role in the pathophysiology of COVID-19, as viral entry into host cells is dependent on its presence [354]. Importantly, ACE2 receptors are present throughout the ileum and colon, in addition to other organs such as the lung, kidneys, and heart [355], and receptor regulation is interrelated with SCFA production [356]. Study findings have shown that SCFA-treated mice had decreased viral activity both in the airways and intestines through the downregulation of ACE2 receptor expression [356]. Further, the luminal activation of the ACE2 receptor by the COVID-19 virus upregulates its expression, leading to reduced gut barrier integrity and leaky gut syndrome. In turn, this can promote metabolic endotoxemia [357] through LPS production, particularly worsened in a pre-existing inflammatory state such as those seen in T2DM patients [358]. It has been demonstrated that healthy plant-based foods such as fruits or vegetables, rich in SCFA-producing and antioxidant capacity, are associated with a lower severity of COVID-19 infection [359], potentially in part through the inverse correlation of inflammatory microbial species including *Escherichia* [360] in combination with the microbiota-dependent mechanisms described above. 

On the other hand, TMAO, found primarily in red meats, is shown to promote the overproduction of IL-6, thereby infecting human endothelial progenitor cells and worsening disease severity [361]. In individuals with diabetes, elevated IL-6 levels at the time of hospitalization were significantly related to early mortality risk [362]. Interestingly, omega-3 PUFA supplementation, an essential component of the Mediterranean diet, was shown to directly inhibit these negative pro-inflammatory effects of TMAO [362]. These beneficial changes occurred through the inactivation of the NF-Kβ signaling pathway as well as a decreasing expression of ACE2, an important regulator in COVID-19 disease severity [361]. Taken together, these findings suggest that nutrition may serve an important role in regulating pathophysiologic changes in COVID-19 outcome through the optimization of microbiota-related metabolites [350]. Therefore, improving dietary intake in individuals with chronic inflammatory diseases such as those with T2DM may improve outcomes when affected by COVID-19.

## 8. Conclusions and Perspective

Over the years, substantial evidence has accumulated supporting the influence of dietary changes in modulating gut microbiota in ways that safeguard against or contribute to the development of T2DM. In this review, we identified the roles of key macronutrients, micronutrients, and various food groups in these processes. Particularly, we showed the effects of important microbiota metabolites including SCFAs, BCAAs, TMAO, secondary bile acids, gut hormones, and inflammatory signaling and how nutrients that are associated with the development of T2DM have distinct metabolite profiles that mechanistically lead to or protect against insulin resistance. In general, SCFA-producing species such as *Lactobacillus*, *Faecalibacterium*, *Akkermansia*, and *Eubacterium*, are induced by favorable nutrients such as cereals, nuts, oilseeds, fruits, and vegetables, while inflammatory species such as *Escherichia coli*, *Ruminococcus torques*, and *Bacteroides* spp. are associated with red meats, fats, and sugary foods. The various food groups discussed are components of common diets such as the MD and WD, which are also shown to have contrasting effects on gut microbiota and the development of T2DM. Through important trends, the mechanisms and associations have been described regarding this topic; the relationship between nutrients, gut microbiota, hyperglycemia, insulin resistance, and the host is complex and new insights are constantly changing our understanding of these processes. Limitations within the studies presented also exist. An increasing number of human studies have elucidated the mechanisms described within this review, though animal studies serve as important models in demonstrating the associations with gut microbiota, their metabolites, and insulin resistance given that 90% of the gut microbiota between mice and humans are deemed to be similar [363]. Still, these murine studies may not translate fully in humans; therefore, the interpretation should be taken with caution when attempting to generalize these findings. Nevertheless, there is significant data showing that gut microbiota is heavily involved at the intersection of nutrition and T2DM.

## Figures and Tables

**Table 1:** Food groups and resulting effects on T2DM.

Food Group	Study Period	Outcome Measured	Results/Implications	Subject Type	Reference
Cereals and Cereal Products	Meta-Analysis	Diabetes risk	Two servings of whole grains decreased the risk of developing T2DM by 21% Refined grain intake increased the risk of developing T2DM by 6–14%	Humans	[272]
	3 months	Metabolic Parameters	Improvements in lipid quality, HgbA1c, BMI, adipose distribution, and fasting C-peptide levels	Humans	[263]
	6 weeks	Gut Microbiota and Inflammatory Markers	Whole grains improved effector memory T-cell activity and acute innate immune response Increased quantity of SCFAs and SCFA-producing genera including *Lachnospira* Decreased relative abundances of pro-inflammatory bacterial family *Enterobacteriaceae*	Humans	[275]
	9 weeks	Gut Microbiota and Gut Hormones	Increased SCFA-producing species Increased GLP-1 secretion	Mice	[278]
	12 weeks	Gut Microbiota	Increased *Bifidobacterium* and decreased *Bacteroides*	Humans	[279]
Fruits and Vegetables	Meta-Analysis	Diabetes risk	Intake of 0.2 servings per day of green leafy vegetables reduced the risk for type 2 diabetes by 13%	Humans	[284]
		Gut Microbiota and Metabolic Parameters	Increased *Akkermansia* Reduced *Lachnoclostridium*, *Desulfovibrio*, *Colidextribacter,* and *Blautia* Upregulation of lipolysis through the FXR, bile acid metabolism pathway	Mice	[292]
		Inflammatory Markers and Metabolic Parameters	Reduced LPS/TLR-4 activity, TNF-α, and IL-6 Improved IL-10 Improved insulin resistance and HgbA1c Increased GLP-1 secretion	Mice	[293]
		Glucose Metabolism and Gut Microbiota	Upregulation of the IRS/AKT signaling pathway to increase GLUT4 translocation and synthesis of glycogen Improved Firmicutes-to-Bacteroidetes ratio	Mice	[295]
Milk and Dairy Products		Diabetes risk	One serving of dairy per day has beneficial effects on T2DM risk reduction of 9% in men and 4% in women	Humans	[310,311]
	3 weeks	Gut Microbiota	Increased *Bifidobacterium* and *Lactobacillus* spp. Increased serum IgA Decreased *Bacteroides fragilis*	Humans	[306,312]
		Gut Microbiota and Pancreatic Function	*Lactobacillus* isolated from yogurt increased SCFA levels and SCFA receptors, GPR41/43 Increased SCFA-producing genera Inhibited reduction of β-cell mass	Mice	[315]
Meat and Meat Products	Meta-Analysis	Diabetes Risk	Risk for T2DM is increased with intake of 100 g of red meat per day Risk for T2DM is increased with intake of 50 g of processed meat per day	Humans	[272,320]
		Gut Microbiota	Red meat decreases *Lactobacillus*, *Paralactobacillus*, and *Prevotella*, while also decreasing SCFAs	Dogs	[327]
	1–4 weeks	Gut Microbiota	Increased *Clostridium* and *Blautia* Decreased *Bifidobacterium* and *Akkermansia*	Mice	[328]
	3 months	Gut Microbiota, Inflammatory and Metabolic parameters	Pork meat decreased *Blautia*, *Bifidobacterium*, and *Alistipes* Induced low-grade inflammation Induced oxidative stress Upregulated lipid metabolism genes including PPAR-α and PPAR-γ	Mice	[332]
Nuts, Oils and Oilseeds	3 months	Parameters of T2DM	Peanuts or almonds in patients with T2DM improved blood glucose, HgbA1c, and inflammatory markers like IL-6 expression	Humans	[335]
	6 weeks	Gut Microbiota and Metabolic Parameters	Nut intake increased the abundance of *Faecalibacterium*, *Clostridium*, *Dialister,* and *Roseburia* and decreased the abundance of Ruminococcus, *Dorea*, *Oscillopira,* and *Bifidobacterium* Decreased pro-inflammatory bile acid production and LDL cholesterol	Humans	[337]
	5 weeks	Gut Microbiota and Inflammatory Parameters	Dietary flaxseed oil decreased severity of T2DM, improved the Firmicutes-to-Bacteroidetes ratio, while increasing *Alistipes* Reduction in IL-1β, TNF-α, IL-6, and LPS production	Rats	[344]

Abbreviations: T2DM, type 2 diabetes mellitus, HgbA1c, Hemoglobin A1c; BMI, body mass index; SCFA, short-chain fatty acid; GLP-1, glucagon-like peptide 1; FXR, Farsenoid X Receptor; LPS, lipopolysaccharides; TLR-4, Toll-like receptor 4; TNF-α, Tumor Necrosis Factor alpha; IL, interleukin; GLUT4, Glucose Transporter 4; IRS, insulin receptor substrate; IgA, Immunoglobulin A; PPAR, peroxisome proliferator-activated receptor; LDL, low density lipoprotein.
